# Correlation between concurrent activation potentiation of hand grip strength and core stability endurance in medical university students

**DOI:** 10.12669/pjms.42.2.12481

**Published:** 2026-02

**Authors:** Nida Waheed, Muhammad Khan, Bhoomika Vassu, Simran Sachrani, Pirya Kumari

**Affiliations:** 1 Dr. Nida Waheed (PT), Institute of Physical Therapy and Rehabilitation, Jinnah Sindh Medical University, Karachi, Pakistan; 2 Dr. Muhammad Khan (PT), Institute of Physical Therapy and Rehabilitation, Jinnah Sindh Medical University, Karachi, Pakistan; 3 Dr. Bhoomika Vassu (PT), Institute of Physical Therapy and Rehabilitation, Jinnah Sindh Medical University, Karachi, Pakistan; 4 Dr. Simran Sachrani (PT), Institute of Physical Therapy and Rehabilitation, Jinnah Sindh Medical University, Karachi, Pakistan; 5 Dr. Pirya Kumari (PT), Institute of Physical Therapy and Rehabilitation, Jinnah Sindh Medical University, Karachi, Pakistan

**Keywords:** Core stability, Concurrent activation, Hand dynamometer, Handgrip strength, Pressure biofeedback unit

## Abstract

**Background & Objectives:**

Concurrent activation potentiation (CAP) is a phenomenon of activation of one muscle area leading to activation of other body areas such as teeth clenching enhances the activity of neck and core stability muscles. The aim of this study was to investigate the effect of hand grip activation on the activation of core stability muscles and to determine the concurrent activation potentiation between hand grip strength and core stability muscles in medical university students.

**Methodology:**

This study entails a cross-sectional research design that was been conducted at the Institute of Physical Therapy and Rehabilitation Jinnah Sindh Medical University, Karachi from January 2024 to March 2024. A total of 196 medical university students were recruited for the study. The data was collected under three conditions that is assessment of handgrip strength of dominant hand, assessment of core stability endurance and assessment of core stability with handgrip. Hand dynamometer and Pressure Biofeedback Unit was used to collect the data. A p-value of 0.05 was considered significant.

**Results:**

The results revealed limited concurrent activation potentiation between handgrip and core stability endurance (mean Pressure Biofeedback Unit score 65.22 without hand grip and 65.28 with hand grip). Furthermore, a significant correlation (p < 0.05) between hand grip strength and concurrent core stability muscle endurance was found (r = 0.591), indicating a moderate and positive relationship.

**Conclusion:**

The correlation between concurrent activation potentiation of hand grip strength and core stability muscle endurance is moderate and positive. Further research is required to clarify the correlation between concurrent activation potentiation of handgrip strength and core stability muscle endurance in young adults.

## INTRODUCTION

Concurrent activation potentiation (CAP) refers to the phenomenon where activities performed in one area activate muscles in the remote areas of the body, resulting in an enhancement of performance.^1^ CAP can lead to increased strength, power, or endurance compared to performing the activities separately.^2^ Teeth clenching may enhance the performance of core muscles, often referred to as the trunk, or the area that connects the legs and arms, plays a crucial role in CAP.^3^ Core muscles provide essential support and control during dynamic movements, helping to stabilize the spine and transfer forces efficiently throughout the body. Core workouts are designed to stretch and strengthen these muscles, improving their ability to control the spine and enhance overall movement performance.^4^ Several studies have established the relationship between teeth clenching and core muscle activation that justifies the phenomenon on CAP.^5^,^6^ In addition, Olsson et al.^7^ studied the correlation between core endurance, handgrip strength and spinal mobility in horseback riders and soccer players. In this study endurance time of the trunk extensor test and the lateral flexor tests both had a moderately positive association with handgrip strength. However, another study showed only a marginal relationship between the strength of the grip with dominant and non-dominant hands and the activation of the core muscles.^8^

Previous studies have established weak relationship of hand grip strength with core muscle strength.^8^,^9^ Even though the plethora of studies examining various aspects of grip strength, clenching of the teeth, and abdominal muscular contractions, there remains a conspicuous absence of research specifically exploring the concurrent activation of core muscles with hand grip.^10^ Thus the aim of this study was to investigate the correlation between concurrent activation potentiation of hand grip strength and core stability endurance in medical university students. The findings from this study may inform the development of targeted interventions aimed at improving hand grip strength and core muscle stability endurance in younger population, thereby potentially mitigating the risk of low back pain.^11^

## METHODOLOGY

The cross-sectional study was conducted at the institute of physical therapy and rehabilitation Jinnah Sindh Medical University Karachi from January 2024 to March 2024.

By using non probability convenience sampling technique 196 (145 females and 51 males) medical students were recruited for the study. The sample size was calculated by using an open Epi sample size calculator taking confidence interval 50% with margin of error as 7.0%, then the estimated sample size was n = 196.^8^

### Inclusion and Exclusion Criteria:

The inclusion criteria were both male and female medical students between the ages of 19-29 years. The exclusion criteria were participants having cervical radiculopathy, any upper limb injury trauma or musculoskeletal condition affecting grip and core strength, neurological symptoms such as numbness and tingling sensations and upper and lower limbs, pregnancy, complaint of generalized weakness in upper limbs.

### Ethical approval:

It was obtained from the Ethical review board of Jinnah Sindh Medical university with IRB Ref. No: JSMU/IRB/2023/784; dated: November 13, 2023.

### Data collection:

Written informed consent was obtained from all the participants who fulfilled the inclusion criteria. The participants were provided with a form containing details about their demographic details. The procedure was explained to the participants so that they could develop a better understanding for the two tools which we had used, Hand dynamometer and Pressure Biofeedback unit. The participants were informed that they could withdraw from the study without any reason if they do not wish to participate further. The data was collected under three conditions that is assessment of handgrip strength of dominant hand, assessment of core stability endurance and assessment of core stability with handgrip. For handgrip assessment the participants held a standing position with shoulder adducted and neutrally rotated, Elbow flexion at 90°, Forearm in mid pronation and unsupported position while wrist is in neutral to 30° extension. While maintaining this position of the body the participants were asked to hold a dynamometer maintaining in their dominant hand and squeeze the dynamometer handle to their maximal strength and sustain it for five seconds followed by a resting period of 15 to 20 seconds. The average of three readings were recorded for each participant.^8^ The core stability endurance was assessed by using pressure biofeedback unit in upright standing and wearing a lumbar belt around the abdomen to hold the apparatus. Pressure bag of the apparatus was placed under the lumbar support just above the anterior superior iliac spine. Pressure bag was then pumped at 70mm Hg and the participants were instructed to perform abdominal drawing by pulling the abdominal wall inside and hold the position for five seconds during which pressure drop readings in the apparatus were taken.^12^ The average of the three readings were recorded for each participant. For the concurrent assessment of core stability endurance with hand grip the participants were instructed to make handgrip and concurrently perform abdominal drawing in standing position as above. The average of three readings were recorded for each participant.

### Statistical Analysis:

Data was analyzed using SPSS version 26. Pearson Correlation method was used to find concurrent activation between hand grip and core stability muscle activation. A p-value of <0.05 was considered significant.

## RESULTS

Overall, 196 subjects participated in the study. Detailed demographic details of the participants are given in Table-I and II. The age range of the students, mean age, median age, core stability (PBU), hand dynamometer score and the hand grip + core stability PBU score have been shown in Table-III.

**Table-I:** Frequency Distribution of Gender.

Gender	N	%
Female	145	74.00%
Male	51	26.00%
Total	196	100.00%

**Table-II:** Frequency Distribution of Dominant Hand.

Which hand is your dominant one?	N	%
Left	23	11.70%
Right	173	88.30%
Total	196	100.00%

**Table-III:** Descriptive Statistics of Age, Core Stability (PBU), Hand Dynamometer and PBU.

Statistics	Age	PBU (mmHg)	Hand Dynamometer (Pounds)	Hand Grip + PBU
N	196	196	196	196
Mean	21.61	65.22	20.10	65.28
Median	22	65.33	20.42	66
Std. Deviation	1.86	2.29	6.38	2.50
Range	10	13.33	31.9	14.66
Minimum	19	56	7.17	54.67
Maximum	29	69.33	39.07	69.33

The Table-IV shows that the p-value of the correlation coefficient between Core stability (PBU) and Hand Grip + Core stability (PBU) was 0 considered as 0.001, which is less than 0.05 therefore; it is concluded that there is significant relationship between Core stability (PBU) and Hand Grip + Core stability (PBU). The correlation of r=0.591 shows the moderate and positive correlation between core stability (PBU) and Hand grip + Core stability (PBU).

**Table-IV:** Correlation Coefficient between PBU (Core Stability) and Hand Grip.

Correlations		Core Stability (PBU)	Hand Grip + (PBU)
Core Stability (PBU)	Pearson Correlation	1	.591**
	Sig. (2-tailed)		0
	N	196	196
Hand Grip + (PBU)	Pearson Correlation	.591**	1
	Sig. (2-tailed)	0	
	N	196	196

## DISCUSSION

To the best of author’s knowledge this is the first study to examine concurrent activation between hand grip and core stability muscles. The findings of this study have suggested significant correlation between concurrent hand grip and core stability muscles activation. It is a common phenomenon that during sport activities or heavy lifting athletes clench their teeth to generate maximum strength in their limbs known as concurrent activation potentiation (CAP).^13^ Therefore, this study was based on the concept of CAP to study the concurrent activation between hand grip and core stability muscles. In the present study the correlation coefficient between the pressure biofeedback unit measuring core stability muscle endurance and hand grip was r = 0.591 and (P-value less than 0.05) indicating significant correlation between hand grip and core stability muscle endurance. The results of another study involving physiotherapists found only a marginal relationship between grip strength and core muscle activation when assessing the grip strength and core stability muscles endurance separately suggesting the need for further investigation.^8^ In the present study the focus was to investigate the CAP between hand grip and core muscles activation and the promising results highlighted the need for further studies. Both of these studies included participants of younger age 18 to 25 years^8^ and 25 to 29 years in the present study showing similarity of age in studied population. Osteopenia is common in younger age group especially females^14^,^15^ and grip strength is an independent predictor of bone mineral density in both genders.^16^ Future studies in younger population may need to consider osteopenia factor in the assessment of hand grip.

The present study and Solanki et al.^8^ utilized same outcome measures that is handgrip dynamometers and pressure biofeedback unit. In both clinical and research contexts, handgrip dynamometers are commonly utilized to assess handgrip strength.^17^ Hand grip strength is frequently employed as a measure of total physical strength, reflecting the performance of hand and forearm muscles. It is widely recognized as an objective indicator of upper extremity functional integrity and is often used to evaluate the efficacy of treatment approaches.^8^,^9^ Similarly pressure biofeedback unit has been reported as a reliable outcome measure to assess core stability muscle endurance.^18^ According to the literature, at least 4 mm Hg to 5.82 mm Hg drop in the pressure gauge of the biofeedback unit shows good abdominal muscle activity.^19^ In the current study, pressure biofeedback median values were 65.33 to 66 mm Hg showing good core stability endurance and corresponds to the scores of clinical significance.

Another study on the correlation between core stability and hand grip strength found weak correlation between hand grip and core strength in cricket players.^9^ One of the interesting findings of the study is that this study assessed both dominant and non-dominant hand grip correlation with core stability muscles. Whereas, the present study only assessed the correlation with dominant hand grip. The results of Akbulutt’s et al.^4^ study supports the findings of the present study in which core training program among sports science students found positive effects on handgrip strength but no significant difference between the training and control groups, pointing to potential confounding factors that warrant further investigation.

The findings of a recent cross-sectional study on young undergraduate students have demonstrated positive relationship between core endurance, hand grip strength, and reaction time. In this study only included 52 young participants therefore, it is difficult to generalize the results to a wider population.^20^

The exact phenomenon by which hand grip strength can influence concurrent activation of core muscles is unknown. However, during the handgrip test, autonomic nerve responses are triggered by muscle contraction in the hand. These responses may vary depending on the activated muscle groups and their locations, leading to various physiological reactions.^21^

### Limitations:

The study used pressure biofeedback unit to measure core muscle endurance which was somewhat difficult to explain to participants. Future studies may include electromyography or ultrasound imaging to measure the activity of the core muscles.

## CONCLUSION

This study found a significant positive correlation (r=0.591, p<0.05) between hand grip strength and core stability concurrent activation in medical university students, measured with hand dynamometry and pressure biofeedback units. Further research is required to clarify these findings further.

## References

[1] Issurin VB, Verbitsky O (2013). Concurrent activation potentiation enhances performance of swimming race start. Acta Kinesiologiae Universitatis Tartuensis.

[2] Allen C (2019). Concurrent activation potentiation-inconsequential event or viable ergogenic strategy. NSCA Coach.

[3] Khan M, Zafar H, Gilani SA, Farooqui WA, Ahmad A (2024). The effects of lumbar stabilization exercises with and without jaw movements in non-specific low back pain (A randomized controlled trial). Pak J Med Sci.

[4] Akbulut T, Çınar V, Söver C, Karaman M (2020). Investigation of effects four-week core training program on some physical parameters. Gaziantep Üniversitesi Spor Bilimleri Dergisi.

[5] Alghadir AH, Zafar H, Iqbal ZA (2015). Effect of three different jaw positions on postural stability during standing. Functl Neurol.

[6] Hirabayashi R, Edama M, Saito A, Yamada Y, Nawa R, Onishi H (2022). Effects of clenching strength on exercise performance: verification using spinal function assessments. Sports Health.

[7] Olsson M (2020). Core endurance and correlation to spinal rotation mobility and hand grip strength in female horseback riders and soccer players.

[8] Solanki DV, Soni N (2021). Correlation between Hand Grip Strength and Core Muscle Activation in Physical Therapists of Gujarat. Int J Health Sci Res.

[9] Sathya P, Dudekula S (2015). Correlation between Hand Grip Strength and Core Muscle Strength in Cricket Players. Int J Sci Res.

[10] Huat Foo L, Zhang Q, Zhu K, Ma G, Greenfield H, Fraser DR (2007). Influence of body composition, muscle strength, diet and physical activity on total body and forearm bone mass in Chinese adolescent girls. Br J Nutr.

[11] Choi S, Nah S, Jang H, Moon J, Han S (2021). Association between relative handgrip strength and chronic lower back pain: a nationwide cross-sectional analysis of the Korea National Health and nutrition examination survey. Int J Environ Res Public Health.

[12] Khan M, Zafar H, Gilani SA (2022). Inter-rater reliability of pressure biofeedback unit among individuals with and without chronic low back pain. Pak J Med Sci.

[13] Ebben WP, Flanagan EP, Jensen RL (2008). Jaw clenching results in concurrent activation potentiation during the countermovement jump. J Strength Cond Res.

[14] Soomro RR, Ahmed SI, Khan M (2017). Frequency of osteopenia and associated risk factors among young female students. J Pak Med Assoc.

[15] Soomro RR, Ahmed SI, Khan M, Ali SS (2015). Comparing the effects of Osteoporosis Prevention Exercise Protocol (OPEP) versus walking in the prevention of osteoporosis in younger females. Pak J Med Sci.

[16] Chan DC, Lee WT, Lo DH, Leung JC, Kwok AW, Leung PC (2008). Relationship between grip strength and bone mineral density in healthy Hong Kong adolescents. Osteoporos Int.

[17] McGrath R (2021). Are we maximizing the utility of handgrip strength assessments for evaluating muscle function?. Aging Clin Exp Res.

[18] Solanki VD, Soni N (2021). Interrater and Intrarater reliability of pressure biofeedback unit in measurement of transverses abdominis muscle activation in asymptomatic adults. Int J Adv Res.

[19] Cairns MC, Harrison K, Wright C (2000). Pressure biofeedback: a useful tool in the quantification of abdominal muscular dysfunction?. Physiotherapy.

[20] Tekeoğlu Tosun A, Eryıldız E, Işıklar Ç, Tekin De Las Peñas D (2025). The Relationship between Core Endurance, Hand Grip Strength, and Reaction Time in Young Adults. Istanbul Gelisim Univ J Health Sci.

[21] Tapiainen AA, Zaproudina N, Lipponen JA, Tarvainen MP, Vierola A, Rissanen SM (2022). Autonomic responses to tooth clenching and handgrip test. Acta Odontol Scand.

